# Molecular profiling unveils genetic complexity and identifies potential new driver mechanisms in head and neck paragangliomas

**DOI:** 10.1016/j.gendis.2025.101705

**Published:** 2025-06-04

**Authors:** Sara Mellid, Eduardo Caleiras, Ángel M. Martínez-Montes, Alicia Arenas, Scherezade Jiménez, María Monteagudo, Rocío Letón, Roberta Radu, Ruth Álvarez-Díaz, Ester Arroba, Alberto Diaz-Talavera, Natalia Martínez-Puente, Cristina Álvarez-Escolá, Marta Pineda, Milagros Balbín, Fátima Al-Shahrour, Cristina Rodriguez-Antona, Cristina Montero-Conde, Luis J. Leandro-García, Emiliano Honrado, Miguel Soria-Tristán, Mercedes Robledo, Alberto Cascón

**Affiliations:** a Hereditary Endocrine Cancer Group, Spanish National Cancer Research Centre (CNIO), 28029 Madrid, Spain; b Histopathology Core Unit, Spanish National Cancer Research Centre (CNIO), 28029 Madrid, Spain; c Monoclonal Antibodies Core Unit, Spanish National Cancer Research Centre (CNIO), 28029 Madrid, Spain; d Bioinformatics Unit, Spanish National Cancer Research Centre (CNIO), 28029 Madrid, Spain; e Centro de Investigación Biomédica en Red de Enfermedades Raras (CIBERER), 28029 Madrid, Spain; f Department of Endocrinology, La Paz University Hospital, 28046 Madrid, Spain; g Hereditary Cancer Program, Catalan Institute of Oncology, Institut d'Investigació Biomèdica de Bellvitge-IDIBELL-ONCOBELL, L'Hospitalet de Llobregat, Barcelona 08908, Spain; h Centro de Investigación Biomédica en Red Cáncer (CIBERONC), 28029 Madrid, Spain; i Molecular Oncology Laboratory, Instituto Universitario de Oncología del Principado de Asturias, Hospital Universitario Central de Asturias, 33011 Oviedo, Spain; j Pharmacogenomics and Tumor Biomarkers Group, Institute for Biomedical Research Sols-Morreale (CSIC-UAM), 28029 Madrid, Spain; k Anatomical Pathology Service, Hospital of León, 24071 León, Spain; l Oncology Department, Hospital Universitario de Getafe, 28905 Getafe, Spain

Pheochromocytomas and paragangliomas (together PPGLs) are rare neuroendocrine tumors arising from chromaffin cells located in the adrenal medulla and ganglia of the autonomic nervous system, respectively. Although paragangliomas located in the head and neck region (HNPGLs) represent approximately 60% of all paragangliomas,^1^ their genetic basis remains less well understood than that of PPGLs with other locations. Furthermore, HNPGLs have been largely excluded from comprehensive genomic profiling studies, leading to the classification of PPGLs into three molecular clusters: pseudohypoxic (C1), kinase signaling (C2), and Wnt-altered (C3). As a result, our understanding of the molecular basis of these tumors is limited, and the discovery of genes exclusively mutated in HNPGLs, such as DNA methyltransferase 3 alpha (*DNMT3A*),^2^ suggests that unique molecular pathways could be involved in their development. Here, we performed a multi-omic characterization of wild-type (WT) HNPGLs, which revealed the existence of two molecular subgroups: succinate dehydrogenase (SDH)-like and DNMT3A-like. In SDH-like HNPGLs, we identified previously undetected alterations in SDH genes despite their positive SDHB immunohistochemistry (IHC), highlighting the risk of overreliance on this method for genetic diagnosis of HNPGLs.^3^ Tumors within the DNMT3A-like cluster showed molecular characteristics consistent with polycomb repressive complex 2 (PRC2) dysfunctions, and stromal antigen 2 (*STAG2*) emerged as a promising new driver.

The study of the prevalence of mutations in known PPGL-related genes in a large series of PPGL patients with single tumors (*n* = 1021) revealed that patients with HNPGLs had a significantly lower rate of mutations (*p* = 1.1 × 10^−6^) (Table S1). Only 4% of HNPGLs carried somatic mutations compared with 30% in pheochromocytomas, suggesting the presence of unknown somatic events contributing to sporadic HNPGLs. In agreement with previous studies, HNPGLs showed a clear female predominance, with more than twice as many women as men. However, this predominance was solely attributable to HNPGL patients without a known mutation (4.11:1 female-to-male ratio compared with PPGLs elsewhere; *p* = 9.6 × 10^−7^) (Fig. 1A).

**Figure 1:**
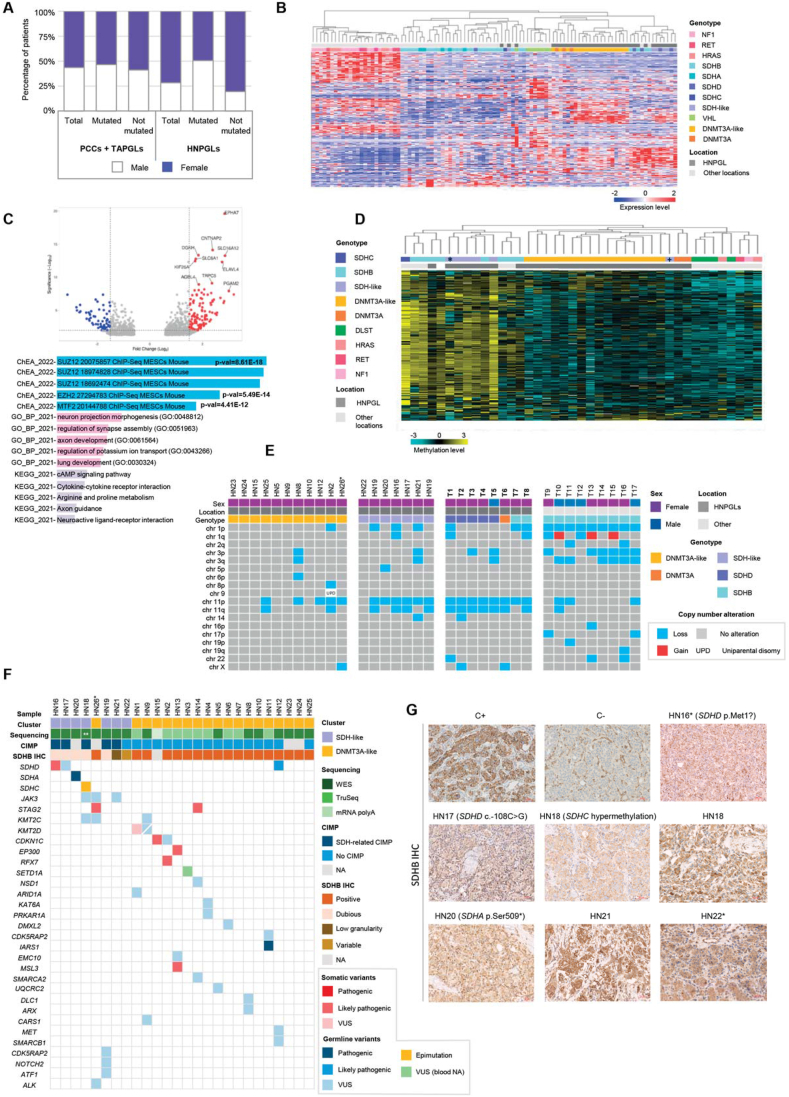
Molecular profiling of head and neck paragangliomas. **(A)** Analysis of patients with single PPGL from our database. Sex distribution of patients according to PPGL location and the presence of mutations in PPGL-related genes. **(B)** Expression profiling of HNPGLs. Unsupervised hierarchical clustering of RNA-sequencing data from a series of 24 WT, 4 *SDHB*-mutated, 3 *SDHD*-mutated, 3 *DNMT3A*-mutated, and 1 *VHL*-mutated HNPGLs, together with previous RNA-sequencing data from representative C1 and C2 tumors in other locations, using a gene signature that distinguishes between PPGL molecular clusters described by Burnichon et al. Two different subgroups of HNPGLs (SDH-like and DNMT3A-like) are evidenced within the pseudohypoxic cluster. **(C)** Differential expression analysis between DNMT3A-like and SDH-like WT HNPGLs. Upper panel, the volcano plot showing 182 differentially expressed genes (Log_2_foldchange < −1.5 or > 1.5; false discovery rate < 0.01) between DNMT3A-like and SDH-like WT HNPGLs. Blue, red, and grey dots represent significantly underexpressed genes, overexpressed genes, and non-significantly differentially expressed genes, respectively. Lower panel, the EnrichR results of significantly enriched pathways related to the differentially expressed genes. The most significant hits include transcription factors SUZ12, EZH2, and MTF2, and biological processes related to nervous system development. **(D)** DNA methylation profile of HNPGLs. Unsupervised clustering of DNA methylation data from available HNPGLs in our series and other representative PPGLs carrying mutations in *DLST*, *DNMT3A*, *HRAS*, *NF1*, *RET*, and SDHx located in the head and neck region and elsewhere, using a signature of differentially methylated probes observed in the CpG island methylator phenotype (CIMP) observed in SDHB mutant renal cell carcinoma and PPGL/GIST. ∗Sample HN17, for which expression cluster assignment was not available due to poor RNA sequencing quality; ^+^Sample HN22, which exhibited an SDH-like transcriptomic profile in the expression profiling analysis. **(E)** Summary of detected copy number alterations. Copy number alterations of available WT HNPGLs corresponding to the DNMT3A-like and SDH-like transcriptomic clusters compared with other representative HNPGLs with known mutations in PPGL-related genes and *SDHB*-mutated PPGLs with other locations (T1-T17 from left to right) are shown. ∗New sample collected from the sister of patient HN18. **(F)** Summary of detected variants. Alterations in SDH genes, chromatin remodeling genes, and other cancer-associated genes identified in the WT HNPGL cohort through whole-exome sequencing, exome capture RNA sequencing, and DNA methylation analysis are shown. Pathogenicity prediction was conducted using the Franklin online tool. Cluster, corresponding group according to transcriptomic profile; CIMP, CpG island methylator phenotype; VUS, variant of uncertain significance. ∗New sample collected from a sister of patient HN18. ∗∗Whole-exome sequencing was conducted on a blood sample from the patient. **(G)** Re-evaluation of SDHB IHC staining in the WT HNPGL series. Two expert pathologists reclassified the SDHB IHC of the samples HN16, HN17, HN18, HN19, and HN20 as dubious/inconclusive. Additionally, the samples HN21 and HN22 exhibited strong staining but low granularity and variable staining depending on the slide area, respectively. All these samples were classified as SDH-like based on transcriptomic results. C+: positive control of a tumor with positive SDHB IHC staining; C−: negative control of a tumor with negative SDHB IHC staining. ∗Tumor with embolization evidence. PPGLs, pheochromocytomas and paragangliomas; HNPGLs, head and neck paragangliomas; TAPGLs, thoracoabdominal paragangliomas; PCCs, pheochromocytomas; IHC, immunohistochemistry.

Transcriptional profiling of 24 WT HNPGLs (all of them developed by women without known genetic alterations and positive for SDHB IHC) (Table S2), 11 HNPGLs carrying known mutations, and a representative series of C1- and C2-mutated PPGLs from other locations, showed that HNPGLs grouped together with C1 tumors regardless of the mutation (Fig. 1B) and formed a distinct cluster enriched in tumors with this location, evidencing their expression peculiarities. Furthermore, two molecular subgroups were evidenced within WT HNPGLs: 18 grouped together with *DNMT3A*-mutated tumors (DNMT3A-like HNPGLs), while the remaining 6 tumors clustered together with those carrying SDHx mutations (SDH-like HNPGLs). Unsupervised clustering performed exclusively with HNPGLs supported this finding (Fig. S1). The only clinical difference between the two groups was age at diagnosis, which was significantly younger in patients with SDH-like tumors (*p* = 0,03) (Fig. S2). One hundred and eighty-two genes were found differentially expressed between DNMT3A-like and SDH-like HNPGLs (false discovery rate < 0.01; log_2_fold change < −1.5 or log_2_foldchange > 1.5; Table S3 and Fig. 1C). Most of these genes were overexpressed in DNMT3A-like tumors (Fig. S3) and were enriched in targets of transcription factors such as SUZ12, EZH2, or MTF2. These transcription factors are related to the polycomb repressive complex 2 (PRC2), a conserved chromatin remodeling complex involved in the trimethylation of histone H3 lysine 27 (H3K27me3), which regulates the maintenance of cell identity and differentiation through transcriptional repression. PRC2 function is frequently deregulated in different cancers, and the overexpression observed in DNMT3A-like HNPGLs suggests that its function may be compromised in these tumors. Genome-wide DNA methylation profiling using two described methylation signatures clustered DNMT3A-like HNPGLs together with *DNMT3A*-mutated tumors, regardless of the methylation signature (Fig. 1D; Fig. S4). This indicates the absence of an SDH-related CpG island methylator phenotype (CIMP) in these tumors and highlights their similarity to HNPGLs carrying mutations in *DNMT3A*. On the other hand, CIMP was observed in all but one SDH-like HNPGL. The low incidence of chromosomal alterations observed in DNMT3A-like HNPGLs (Fig. 1E) and their older mean age of onset suggest that they are unlikely to be caused by germline mutations in genes requiring LOH as a second hit but rather from somatic alterations.

Despite exhibiting positive SDHB IHC staining at diagnosis, next-generation sequencing and methylome analysis of SDH-like HNPGLs led to the identification of four alterations in SDHx genes (Fig. 1F and Table S4). One of these variants had been previously detected by our routine genetic testing (sample HN17; c.-108C > G in *SDHD*) but was initially classified as a variant of uncertain significance. A variant in the same position was reported to cause a significant reduction in *SDHD* promoter activity and is predicted to disrupt several transcription factor-binding sites (Fig. S5). The remaining three SDHx alterations were not detected by our routine diagnostic analysis for various reasons (Fig. S6): a somatic deletion in the promoter region of *SDHD*, a truncating germline variant in *SDHA*, and hypermethylation of the promoter of *SDHC*. This suggests that a significant percentage of WT HNPGLs (16% in our series) may have alterations in known genes that can be overlooked either due to technical limitations or constraints of the technology employed.

A blind re-evaluation of SDHB IHC stains by two independent pathologists classified all DNMT3A-like samples as positive, while five SDH-like samples, including those with SDHx alterations, were deemed dubious/inconclusive (Fig. 1G). The remaining SDH-like tumors exhibited either strong SDHB staining with low granularity or heterogeneous staining patterns. Several studies have previously described weak diffusely cytoplasmic SDHB staining rather than granular in SDH-mutated PPGLs, predominantly HNPGLs. Pre-surgical embolization of HNPGLs has been proposed to influence IHC properties (ENS@T meeting 2023), though only two of the SDH-like HNPGLs underwent this procedure. These findings indicate the need for a more thorough evaluation of HNPGL SDHB IHCs and caution against the overreliance on the accuracy of SDHB IHC in the clinical setting, particularly in HNPGLs. In SDH-like cases where the SDHB IHC was inconclusive and no mutations were identified, the cause could be a still undetected alteration in the SDH genes.

Next-generation sequencing identified 26 candidate variants in 13 of the 18 DNMT3A-like tumors (Fig. 1F and Table S4), mostly affecting genes associated with chromatin remodeling, epigenetic modifications, and other known cancer genes, with no translocations identified. Notably, a germline variant of uncertain significance and a likely pathogenic somatic variant were found in *CDKN1C*. This cancer-related gene is associated with Beckwith-Wiedemann syndrome, a pediatric overgrowth disorder that involves a predisposition to embryonal tumors and, in some cases, bilateral pheochromocytomas. Two somatic *STAG2* mutations, a splice (Fig. S7) and a frameshift variant (Fig. S8), were found in two DNMT3A-like HNPGLs. *STAG2* is frequently mutated in cancer, and its loss may disrupt PRC2-mediated gene expression regulation since it occupies PRC2-marked regulatory regions.^4^ Interestingly, three additional PPGLs (all of them developed by women) have been found carrying somatic *STAG2* truncating mutations in the absence of alterations in PPGL-related genes (https://www.cbioportal.org/and Flynn et al^5^). These findings suggest that *STAG2* mutations could account for up to 3%–10% of PPGL cases without mutations, indicating its potential role as a driver in PPGL development. The negative STAG2 staining observed in mutated HNPGLs (Fig. S9) further supports this hypothesis, although additional research is needed to confirm STAG2's role. In conclusion, our findings highlight the genetic complexity of HNPGLs and have enabled the identification of a novel omic cluster related to PCR2 dysfunction, providing a foundation for future research on these tumors.

## CRediT authorship contribution statement

**Sara Mellid:** Writing – review & editing, Writing – original draft, Visualization, Validation, Methodology, Formal analysis, Data curation. **Eduardo Caleiras:** Writing – review & editing, Formal analysis. **Ángel M. Martínez-Montes:** Software, Data curation. **Alicia Arenas:** Software, Data curation. **Scherezade Jiménez:** Methodology. **María Monteagudo:** Methodology. **Rocío Letón:** Methodology. **Roberta Radu:** Methodology. **Ruth Álvarez-Díaz:** Software, Data curation. **Ester Arroba:** Software, Data curation. **Alberto Diaz-Talavera:** Methodology. **Natalia Martínez-Puente:** Methodology. **Cristina Álvarez-Escolá:** Resources. **Marta Pineda:** Resources. **Milagros Balbín:** Resources. **Fátima Al-Shahrour:** Software, Data curation. **Cristina Rodriguez-Antona:** Writing – review & editing. **Cristina Montero-Conde:** Writing – review & editing. **Luis J. Leandro-García:** Writing – review & editing. **Emiliano Honrado:** Writing – review & editing, Formal analysis. **Miguel Soria-Tristán:** Resources. **Mercedes Robledo:** Writing – review & editing. **A. Cascón:** Writing – review & editing, Supervision, Resources, Project administration, Funding acquisition, Formal analysis, Conceptualization.

## Ethics declaration

This study was conducted according to the Declaration of Helsinki and has been approved by the Instituto de Salud Carlos III Ethics Committee (CEI PI 93_2022). All samples were collected following institutional ethical protocols, including patients' written informed consent for this purpose.

## Declaration of generative AI and AI-assisted technologies in the writing process

During the preparation of this work, the author(s) used DeepL to improve language and readability. After using this tool/service, the author(s) reviewed and edited the content as needed and take(s) full responsibility for the content of the publication.

## Funding

This work was supported by the Instituto de Salud Carlos III (ISCIII), through the “Acción Estratégica en Salud” (AES) (projects PI22_01490 to A.C. and PI20/01169 to M.R.), cofounded by the European Regional Development Fund (ERDF) and by the Paradifference Foundation (no grant number applicable to M.R.). Sara Mellid was supported by the Spanish Ministry of Science, Innovation and Universities “Formación del Profesorado Universitario- FPU” fellowship with ID number FPU19/04940.

## Conflict of interests

The authors declared no conflict of interests.
