# Supplementary material for: Molecular profiling unveils genetic complexity and identifies potential new driver mechanisms in head and neck paragangliomas
Source: Genes Dis. 2025 Jun 4;13(2):101705. doi: 10.1016/j.gendis.2025.101705 (PMC12596578; doi:10.1016/j.gendis.2025.101705)
Supplement: Multimedia component 1 [file mmc1.docx]

**MATERIALS AND METHODS**

**HNPGL samples**

This study was conducted according to the Declaration of Helsinki and has been approved by the Instituto de Salud Carlos III Ethics Committee (CEI PI 93_2022). All samples were collected following institutional ethical protocols, including patients’ written informed consent for this purpose. All patients had been previously studied using an updated version of a custom gene panel (AmpliSeq Custom DNA Panel, Illumina)^1^ including the main PPGL-related genes: VHL, RET, SDHA, SDHB, SDHC, SDHD, SDHAF2, SDHAF1, MAX, HIF1A (exon 12), EPAS1 (exon 12), TMEM127, HRAS, KRAS, NF1, GOT2, FH, MDH2, SLC25A11, DNMT3A (exon 8), DLST (exon 14), MERTK (exon 17), IDH1, IDH2, CSDE1, EGLN1, EGLN2, BRAF (exon 15), MET (exons 14–21), FGFR1 (exons 12 and 14), KIF1B, CDKN1B, MEN1, PTEN, PRKAR1A, H3-3A and ATRX. In addition, an updated version of the series published by Currás-Freixes et al^1^ was used to compare the frequency of patients with HNPGLs, PGLs located in the thoracic or abdominal region (TAPGLs), and PCCs carrying mutations in PPGL-related genes. To do so and considering that PPGL patients may develop multiple tumors in different locations, we took into consideration only patients developing single tumors. Thus, after excluding patients harboring tumors in multiple locations, we were left with 1,021 patients (82% of the total) of whom 55% developed PCCs and 45% developed PGLs (Table S1). Germline DNA from blood samples was extracted from peripheral-blood leukocytes using the Maxwell® 16 Blood DNA Purification Kit (Promega AS1010). Tumor samples were either formalin-fixed paraffin-embedded (FFPE) or fresh-frozen and stored in OCT (frozen). Genomic DNA from FFPE samples was extracted using the Covaris S2 System (truXTRAC™ FFPE DNA microTUBE Kit, Covaris 520136) and from frozen samples using the NucleoSpin Tissue, Mini kit for DNA from cells and tissue (Macherey-Nagel 740952.50), according to the manufacturers’ instructions. Total RNA extraction was performed from FFPE samples using Maxwell^®^ RSC RNA FFPE Kit (Promega AS1440) and from frozen samples using TRIzol™ Reagent (Thermo Fisher Scientific 15596026), following manufacturers’ protocol.

**RNA Sequencing and hierarchical clustering**

3’ mRNA Sequencing cDNA libraries from 26 WT HNPGLs and 2 HNPGLs carrying *SDHD* mutations were prepared by the Genomics Unit of the CNIO according to vendor’s instructions with "QuantSeq 3‘ mRNA-Seq V2 Library Prep Kit (FWD) for Illumina" (Lexogen, Cat.No. 191) and a UMI Second Strand Synthesis module, using 500 ng of total RNA as starting material. Libraries were applied to an Illumina flow cell for cluster generation and sequenced on the Illumina NextSeq 550 or Illumina NovaSeq X. This data was combined with previously published RNA-seq data from 76 additional PPGLs with known mutations (European Genome-phenome Archive ID: EGAS00001006044)^2^ using ComBat-seq^3^, taking into account batch effects due to the type of analysis, platform, cohorts and the origin of the samples (frozen or FFPE). Hierarchical clusterings of z-scored transcriptomic data were performed with the GeneCluster 2.0 software^4^ and the Morpheus software (https://software.broadinstitute.org/morpheus) using the gene expression signature described by Burnichon et al.^5^ including all of the genes differentially expressed in SDHx, *VHL*, *RET*, *NF1* and *TMEM127* PPGLs.

In order to identify both single nucleotide variants and potential gene fusions in WT HNPGLs, we performed Exome capture RNAseq and Paired-end 150bp RNAseq on FFPE samples that met the minimum quality criteria (HN1, HN2, HN3, HN4, HN6, HN7, HN11, HN13 and HN14) and on a single frozen sample (HN15), respectively. RNA library preparation, transcriptome sequencing and subsequent bioinformatics analysis were conducted by Novogene Co., LTD (Beijing, China). The frozen sample was analyzed using the eukaryotic mRNA library with poly A enrichment service and sequenced on an Illumina NovaSeq 6000, and FFPE samples were analyzed using the TruSeq™ RNA Exome service (Illumina). Fusion gene analysis was performed using the STAR-Fusion software (1.9.0) ^6^.

**Differential expression and enrichment analysis**

Differentially expressed (DE) genes were identified using the DEApp tool ^7^ in a single-factor experiment: DNMT3A-like (n=18) *vs* SDH-like HNPGLs (n=6). The data summarization step was used to filter out genetic features with low counts, with an expression cut-off of 1 counts per million in at least 2 samples. DE analysis was conducted using the DESeq2 method. The VolcanoseR ^8^ tool was used to visualize DE data with a false discovery rate (FDR) lower than 0.01 and a Log2 fold change (FC) lower than -1.5 or greater than 1.5. The EnrichR tool ^9^ was used to perform an enrichment analysis of DE genes highlighting the top 5 terms enriched in the following libraries: Gene Ontology Biological Processes, KEGG 2021 Human and ChEA 2022. In addition, enriched chromatin modifications were observed in the Epigenomics Roadmap HM ChIP-seq and ENCODE Histone Modifications 2015 libraries.

**DNA methylation assays**

Genome-wide DNA methylation analysis was performed at the Centro Nacional de Genotipado (CEGEN-ISCIII) using the Infinium MethylationEPIC BeadChip Kit (Illumina 20087706), as previously described ^10^. The EZ DNA Methylation Kit (Zymo Research D5001) was used to perform DNA bisulfite conversion of WT HNPGL samples, following manufacturer’s instructions. β-values were converted to M-values (M = log((-B)/(B-1))/log(2)) and z-scored in order to perform hierarchical clusterings using the Morpheus software (https://software.broadinstitute.org/morpheus). The list of probes differentially methylated in *DNMT3A*-mutated PPGLs ^11^ and the list of probes associated with the CpG island methylator phenotype (CIMP) observed in *SDHB* mutant PPGL and Renal Cell Carcinoma/GIST ^12^ were used for clustering. Methylation data corresponding to tumor samples carrying alterations in PPGL-related genes was previously deposited under the accession numbers GSE111336, GSE123185 and GSE210809 ^13;14^. PCR assessment and validation of *SDHC* promoter methylation was performed using bisulfite-modified DNA as template and the following primers: FW- AATTGTTTATTTTGTGTTTGGGTAG, RV-CCTTAAAATTATTTTCTCAAAACTC.

**Immunohistochemistry**

SDHB, SDHA and STAG2 IHC staining were performed on 3 μm FFPE sections of WT HNPGLs using an automated immunostaining platform (Ventana Discovery XT, Roche). The slides underwent antigen retrieval using RiboCC (pH6) and blocking of the endogenous peroxidase with 3% hydrogen peroxide. The sections were then incubated with either rabbit polyclonal anti-SDHB antibody (Sigma-Aldrich HPA002868), rabbit polyclonal anti-SDHA antibody (Neo Biotech NB-22-20310) or mouse monoclonal anti-STAG2 antibody (Santa Cruz SC-81852). Afterwards, the appropriate horseradish peroxidase-conjugated visualization system was used, followed by the development of the IHC reaction using 3,30-diaminobenzidine tetrahydrochloride (DAB) (Dako). Nuclei were counterstained with Harrys’s hematoxylin. The slides were then dehydrated, cleared, and mounted with a permanent mounting medium for microscopic evaluation by an expert pathologist (EC).

**Whole exome sequencing**

Whole exome sequencing of HNPGL samples was carried out in the CEGEN-ISCIII using the SureSelectXT Human All Exon V6 + COSMIC target enrichment system or the Illumina DNA Prep with Exome 2.5 Enrichment with the Twist Bioscience for Illumina Exome 2.5 Panel, following manufacturer’s instructions. Briefly, 500 ng of genomic DNA samples were fragmented using a Covaris S2 sharing instrument based on the initial sample’s integrity. Fragmented DNA samples underwent end-repair, dA-tailing, and ligation to Illumina’s adapters treatments. Adapter-ligated libraries were PCR amplified, hybridized to SureSelect Oligo Capture Library Mix, and completed by limited-cycle PCR with Illumina TruSeq primers and KAPA HiFi HotStart DNA pol (Roche KK2501). Sequencing was performed on the NovaSeq X Plus in paired-end 100 bp reads mode, achieving a median target coverage of approximately 200×.

**Variant prioritization**

The variants identified through WES and paired-end RNAseq were manually curated taking into account several factors. These included their allele frequency in the general population (no greater than 0.00004% in the gnomAD database), the impact of the variant on the protein, the read depth, the pathogenicity prediction using the Franklin online tool (https://franklin.genoox.com/clinical-db/home), the existence of gene-related neurodevelopmental conditions, and the biological function of the gene. We decided to use as a threshold for the prioritization of germline variants a frequency (0.00004%) that is higher than the one observed in gnomAD for the most common pathogenic mutation in the PPGL gene with the lowest penetrance (i.e., *SDHB*). A selection of candidate variants was confirmed through PCR amplification and Sanger sequencing in tumor and blood DNA samples to determine whether the mutation was somatic or germline.

**Copy number alteration analysis**

Copy number alterations were detected from WES data using the FACETS algorithm (*cnv-facets* R package) ^15^. SNP-Array was performed on high quality DNA tumor samples using 250ng of tumor DNA. The genome-wide scan was conducted using the Infinium Global Screening Array-24 v3.0 BeadChip (Illumina) in the CEGEN-ISCIII according to the manufacturer’s specifications, and analyzed using the Chromosome Viewer tool in GenomeStudio v2.0 (Illumina). The log-R ratio was used as the metric, which represents the binary logarithm of the ratio between the observed and expected normalized R values for a given SNP.

References

1. Curras-Freixes M, Pineiro-Yanez E, Montero-Conde C, et al. PheoSeq: A Targeted Next-Generation Sequencing Assay for Pheochromocytoma and Paraganglioma Diagnostics. *J Mol Diagn.* 2017;19(4):575-588.

2. Calsina B, Pineiro-Yanez E, Martinez-Montes AM, et al. Genomic and immune landscape Of metastatic pheochromocytoma and paraganglioma. *Nat Commun.* 2023;14(1):1122.

3. Zhang Y, Parmigiani G, Johnson WE. ComBat-seq: batch effect adjustment for RNA-seq count data. *NAR Genom Bioinform.* 2020;2(3):lqaa078.

4. Reich M, Ohm K, Angelo M, Tamayo P, Mesirov JP. GeneCluster 2.0: An advanced toolset for bioarray analysis. *Bioinformatics.* 2004;20:1797–1798.

5. Burnichon N, Vescovo L, Amar L, et al. Integrative genomic analysis reveals somatic mutations in pheochromocytoma and paraganglioma. *Hum Mol Genet.* 2011;20(20):3974-3985.

6. Haas BJ, Dobin A, Li B, Stransky N, Pochet N, Regev A. Accuracy assessment of fusion transcript detection via read-mapping and de novo fusion transcript assembly-based methods. *Genome Biol.* 2019;20(1):213.

7. Li Y, Andrade J. DEApp: an interactive web interface for differential expression analysis of next generation sequence data. *Source Code Biol Med.* 2017;12:2.

8. Goedhart J, Luijsterburg MS. VolcaNoseR – a web app for creating, exploring, labeling and sharing volcano plots. 2020.

9. Xie Z, Bailey A, Kuleshov MV, et al. Gene Set Knowledge Discovery with Enrichr. *Curr Protoc.* 2021;1(3):e90.

10. Bibikova M, Le J, Barnes B, et al. Bibikova, M.; Le, J.; Barnes, B.; Saedinia-Melnyk, S.; Zhou, L.; Shen, R.; Gunderson, K.L. Genome-wide DNA methylation profiling using Infinium(R) assay. Epigenomics 2009, 1, 177–200. *Epigenomics.* 2009;1(1):177-200.

11. Remacha L, Curras-Freixes M, Torres-Ruiz R, et al. Gain-of-function mutations in DNMT3A in patients with paraganglioma. *Genet Med.* 2018;20(12):1644-1651.

12. Ricketts CJ, Killian JK, Vocke CD, et al. Kidney tumors associated with germline mutations of FH and SDHB show a CpG island methylator phenotype (CIMP). *PLoS One.* 2022;17(12):e0278108.

13. Remacha L, Pirman D, Mahoney CE, et al. Recurrent Germline DLST Mutations in Individuals with Multiple Pheochromocytomas and Paragangliomas. *Am J Hum Genet.* 2019;104(4):651-664.

14. Mellid S, Garcia F, Leandro-Garcia LJ, et al. DLST mutations in pheochromocytoma and paraganglioma cause proteome hyposuccinylation and metabolic remodeling. *Cancer Commun (Lond).* 2023;43(7):838-843.

15. Shen R, Seshan VE. FACETS: allele-specific copy number and clonal heterogeneity analysis tool for high-throughput DNA sequencing. *Nucleic Acids Res.* 2016;44(16):e131.


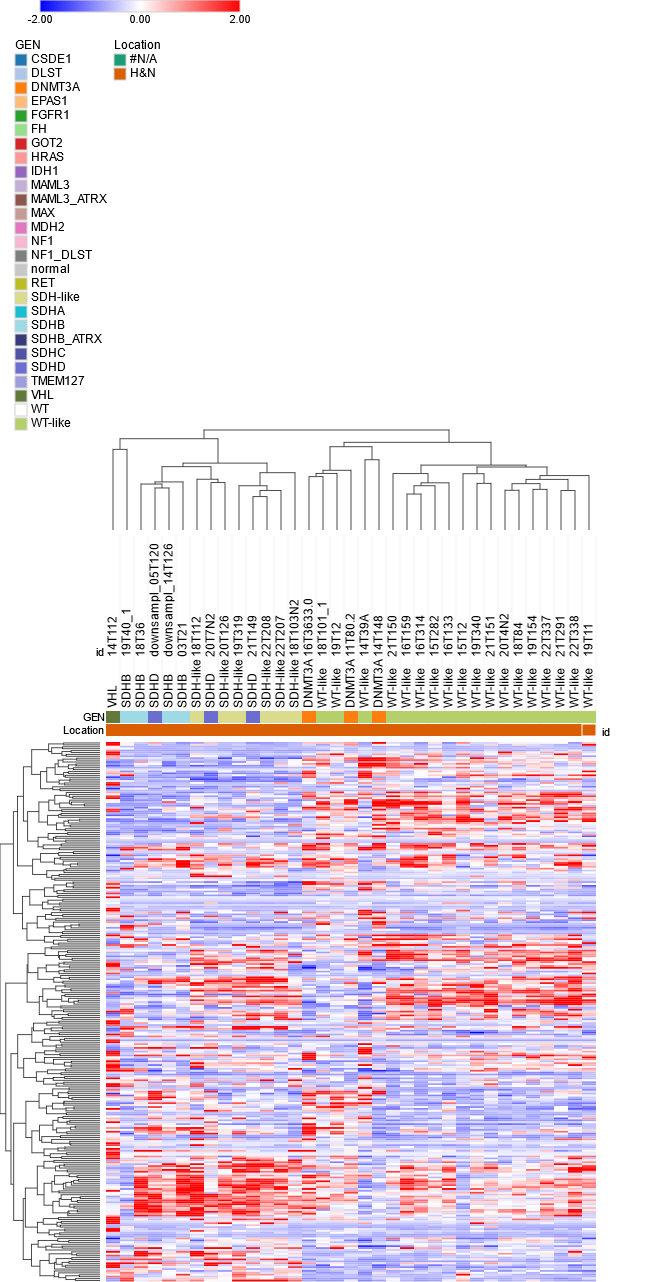


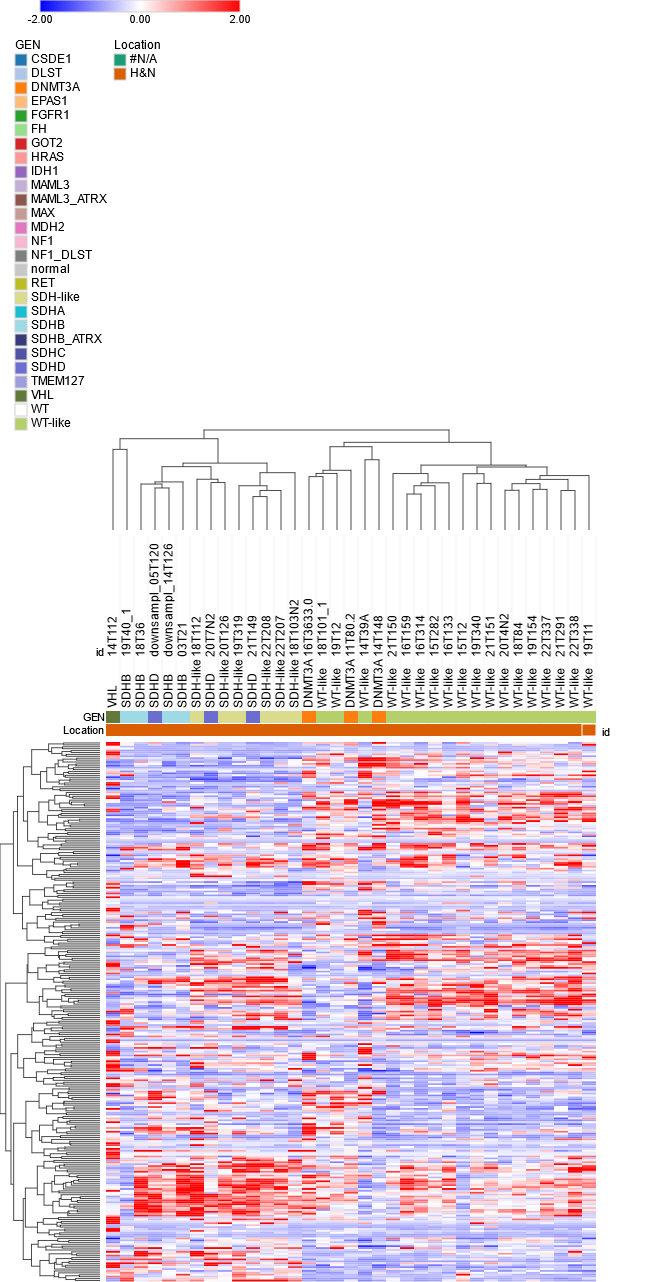


HN8

HN7

HN25

HN5

HN11

HN3

HN4

HN9

HN12

HN15

HN6

HN10

HN1

HN13

HN14

HN2

HN23

HN16

HN18

HN21

HN20

HN22

HN19

HN24

Expression level

SDH-like

DNMT3A

SDHD

VHL

SDHB

DNMT3A-like


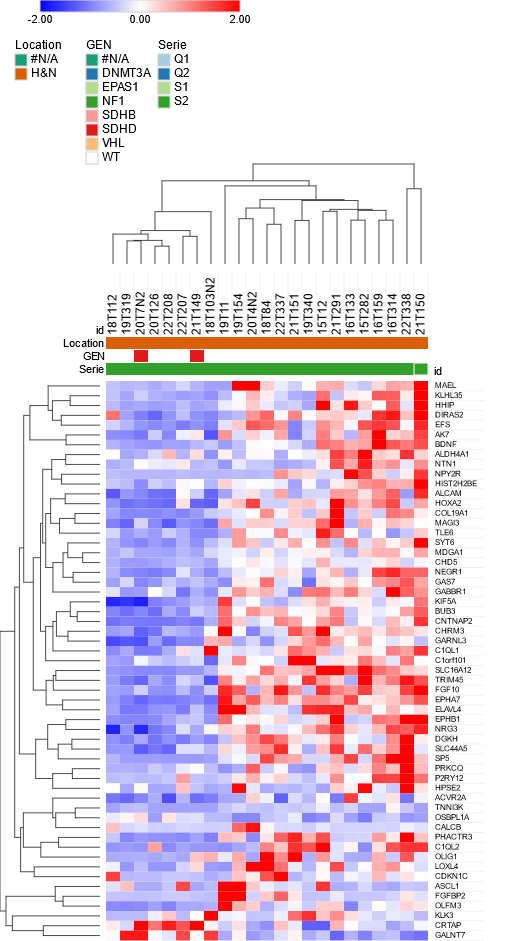


-2

2

0

**Figure S1**. Unsupervised hierarchical clustering of RNAseq data from a series of HNPGLs (24 WT, 4 *SDHB*-mutated, 3 *SDHD*-mutated, 3 *DNMT3A*-mutated and 1 *VHL*-mutated HNPGL) performed using a gene signature that distinguishes between PPGL molecular clusters described by Burnichon *et al.* Two different subgroups of HNPGLs (SDH-like and DNMT3A-like) are evidenced within the pseudohypoxic cluster.


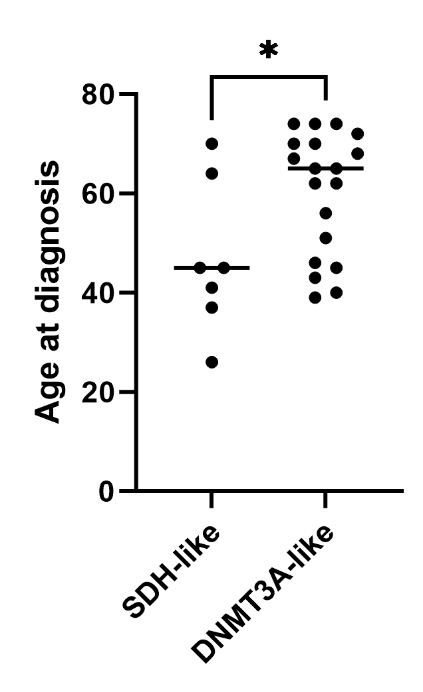


**Figure S2**. Age at diagnosis for patients with WT HNPGLs. Patients with SDH-like HNPGLs exhibited a significantly lower age at diagnosis compared to those with DNMT3A-like tumors (p-value = 0,0313). Bars represent the median of the ages. SDH-like tumors include sample HN17, classified according to its methylation profile. DNMT3A-like tumors include sample HN26, corresponding to the affected sister of patient HN18.


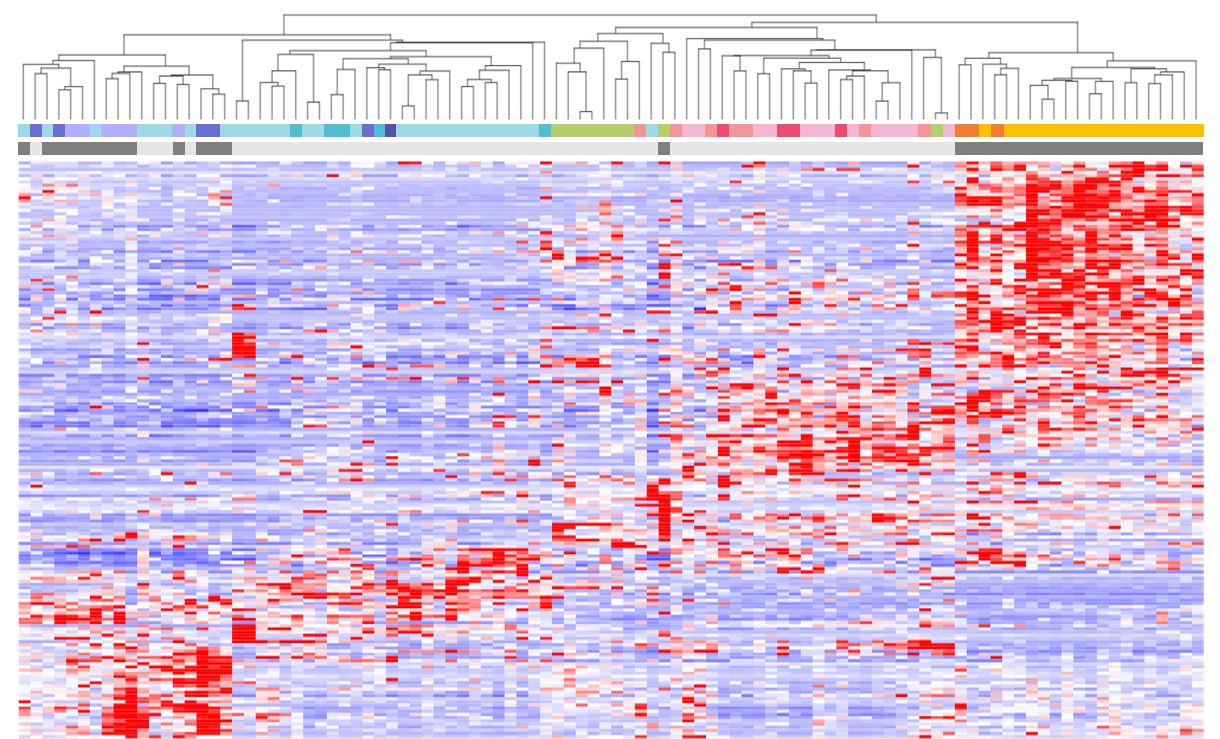


HN8

HN7

HN25

HN5

HN11

HN3

HN14

HN9

HN12

HN15

HN6

HN10

HN1

HN13

HN2

HN23

HN16

HN18

HN21

HN19

HN20

HN22

HN24

HN4

DNMT3A

SDHD

VHL

SDHC

SDHB

SDHA

RET

NF1

HRAS


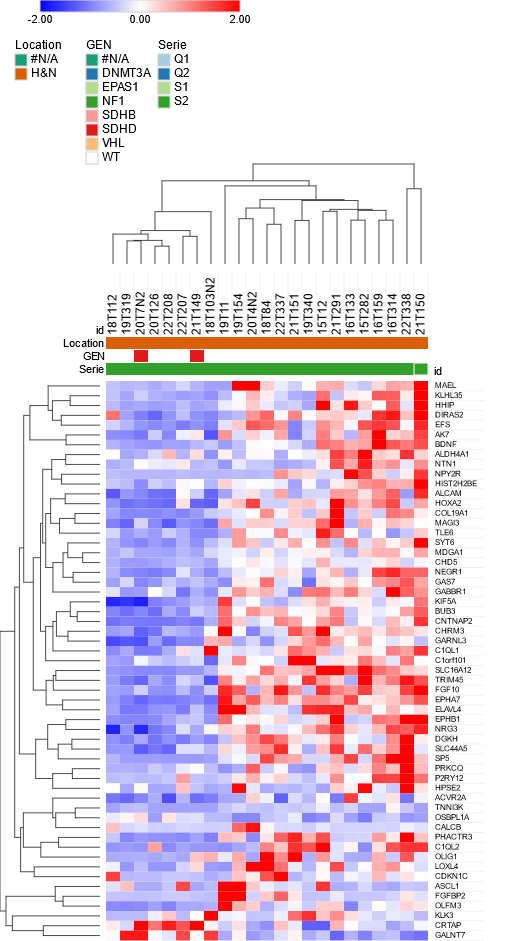


-2

2

0

DNMT3A-like

HNPGL

Other locations

SDH-like

Expression level

**Figure S3**. DE analysis between DNMT3A-like and SDH-like WT HNPGLs. Unsupervised hierarchical clustering of RNAseq data from HNPGLs and representative Clusters 1 and Cluster 2 tumors with other locations, using the list of DE genes as a signature. DNMT3A-like WT HNPGLs show distinct transcriptomic features.


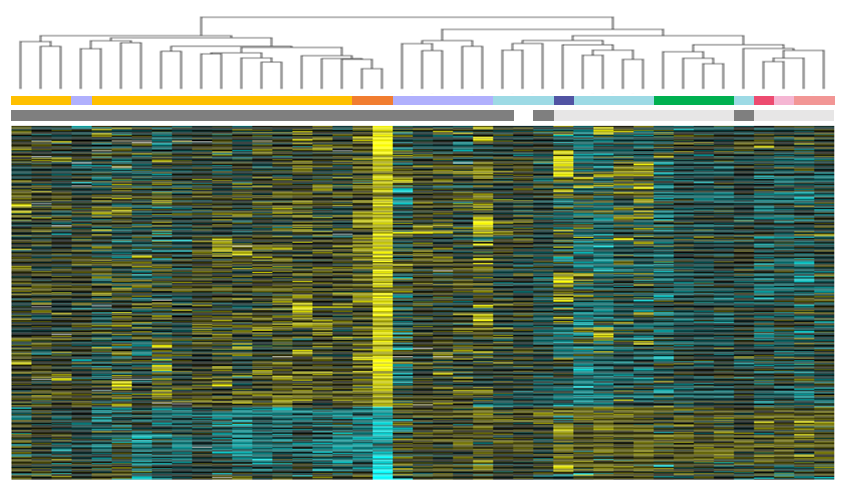


-3

3

0


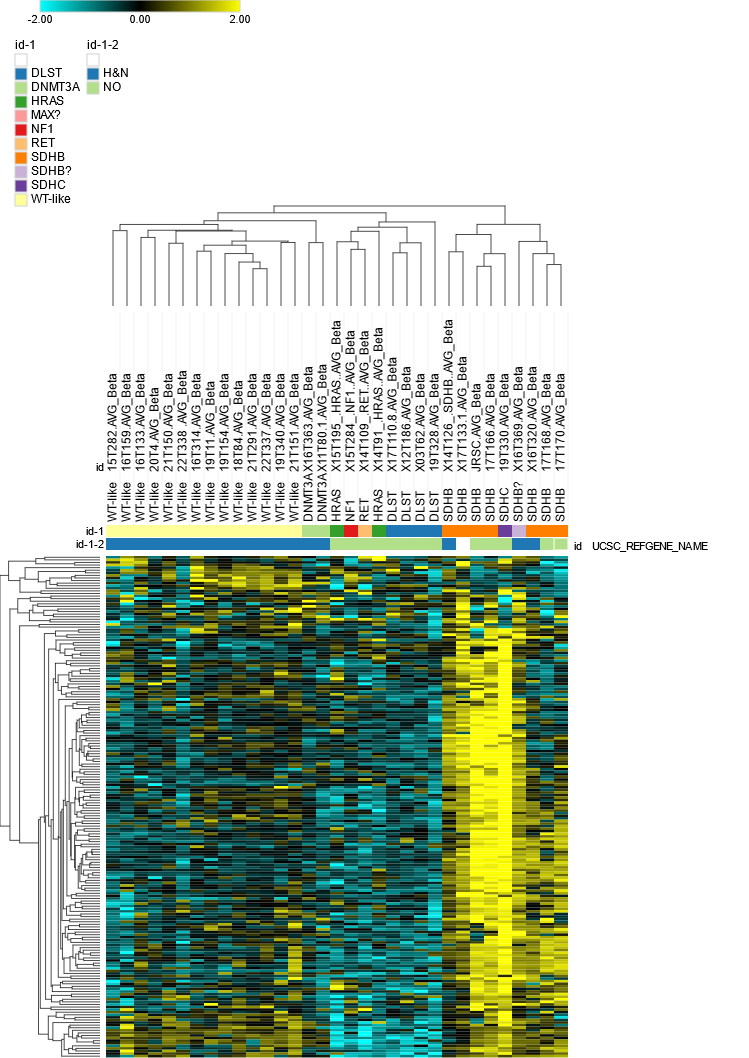


Methylation level

DNMT3A

SDH-like

RET

NF1

DLST

SDHC

SDHB

HNPGL

Other locations

HRAS

DNMT3A-like

HN8

HN7

HN22

HN25

HN5

HN11

HN3

HN14

HN9

HN12

HN15

HN6

HN10

HN1

HN13

HN4

HN2

HN17

HN16

HN18

HN21

HN19

**Figure S4**. Unsupervised clustering of DNA methylation data from available HNPGLs in our series and other representative PPGLs carrying mutations in *DLST*, *DNMT3A*, *HRAS*, *NF1*, *RET*, and SDHx located in the head and neck region and elsewhere, using a list of differentially methylated probes in *DNMT3A*-mutated HNPGLs.


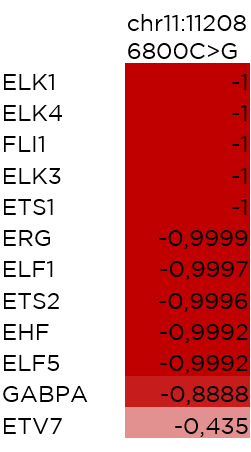


**Figure S5**. Representation of the predicted disruption of several transcription factor binding sites caused by the *SDHD* c.-108 C>T variant using the Fabian tool. Values close to -1 indicate a great likelihood of disruption using transcription factor flexible models and position weight matrices to predict the effect of DNA variants on transcription factor binding.


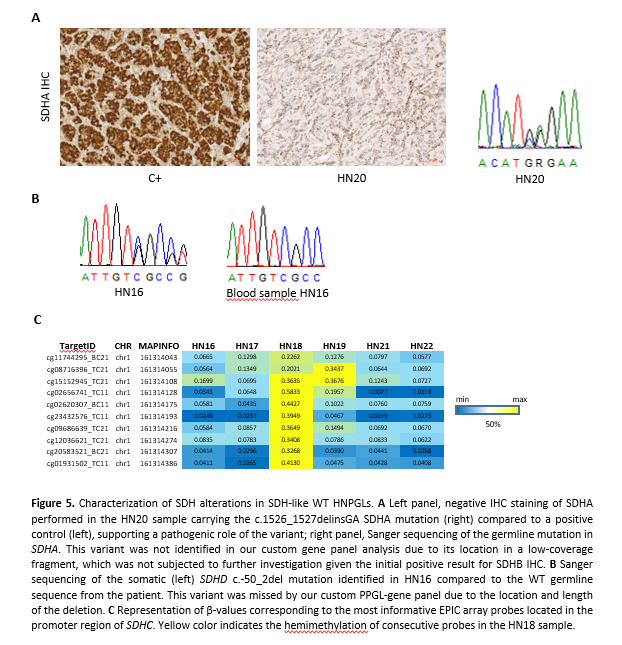

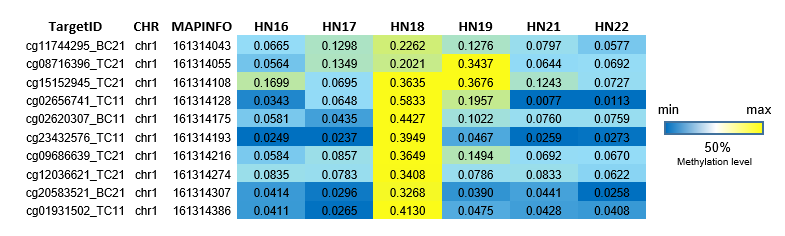

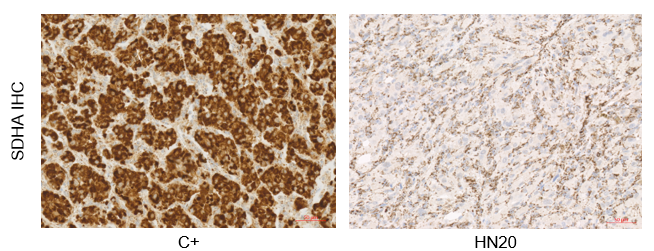

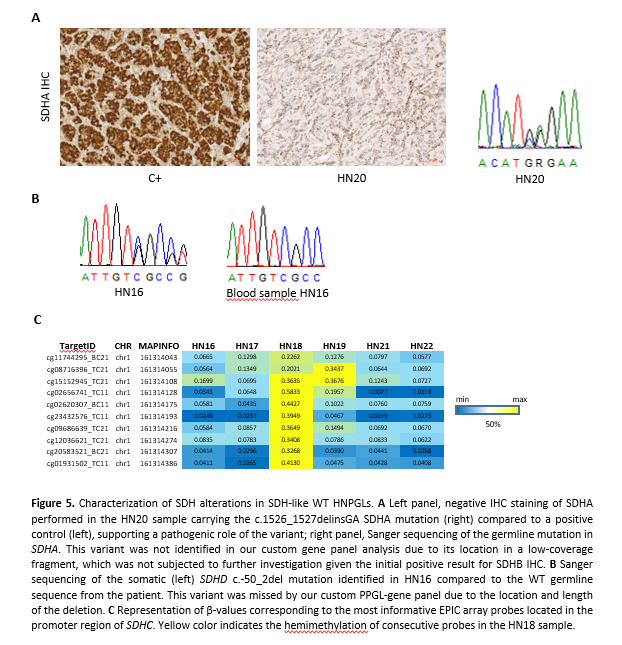


HN16

Blood sample HN16

HN20

**A**

**B**

**C**

**Figure S6**. Characterization of SDH alterations in SDH-like WT HNPGLs. **A** Left panel, negative IHC staining of SDHA performed in the HN20 sample carrying the c.1526_1527delinsGA SDHA mutation (right) compared to a positive control (left), supporting a pathogenic role of the variant; right panel, Sanger sequencing of the germline mutation in *SDHA*. This variant was not identified in our custom gene panel analysis due to its location in a low-coverage fragment, which was not subjected to further investigation given the initial positive result for SDHB IHC. **B** Sanger sequencing of the somatic (left) *SDHD* c.-50_2del mutation identified in HN16 compared to the WT germline sequence from the patient. This variant was missed by our custom PPGL-gene panel due to the location and length of the deletion. **C** Representation of β-values corresponding to the most informative EPIC array probes located in the promoter region of *SDHC*. Yellow color indicates the hemimethylation of consecutive probes in the HN18 sample.


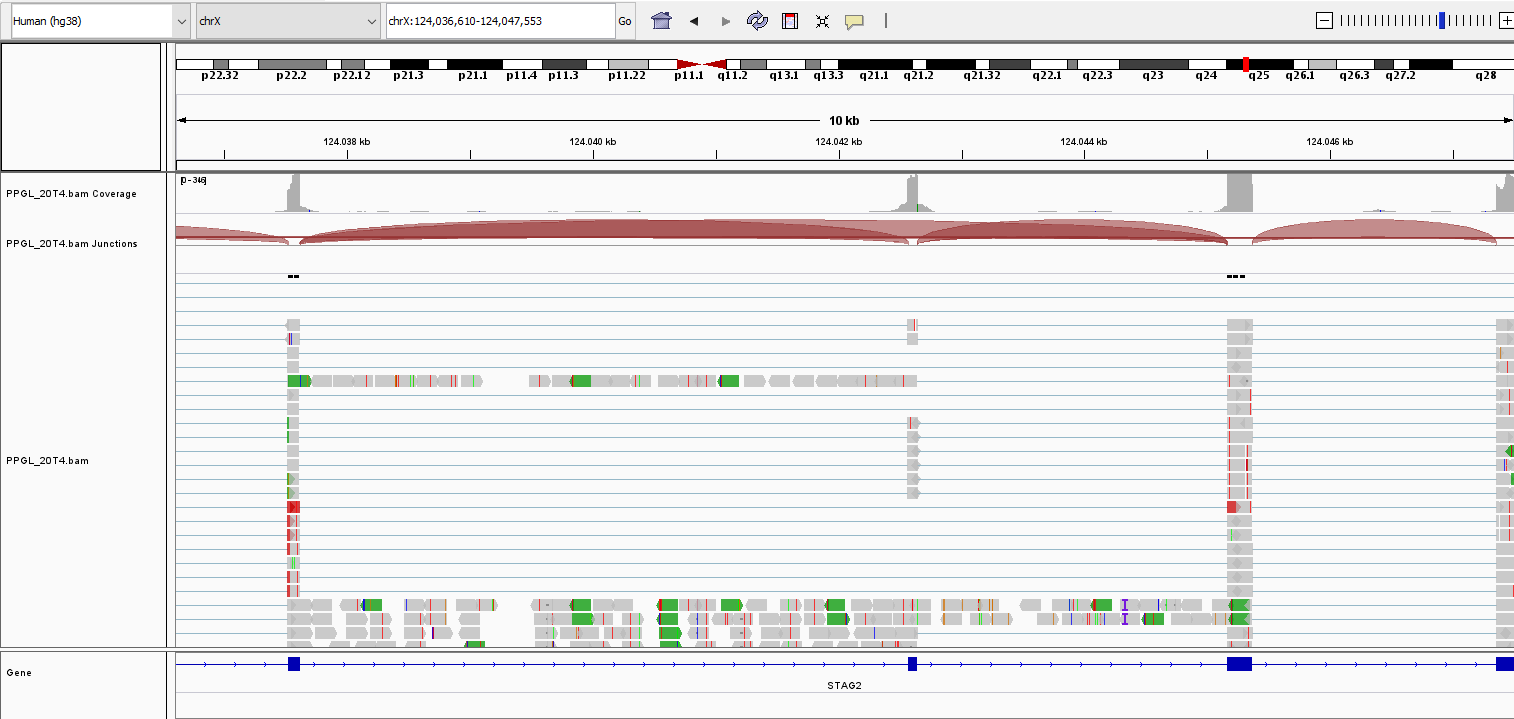


Coverage

Junctions

**Figure S7**. IGV screenshot of exome capture RNAseq data corresponding to the HN14 sample shows the skipping of exon 7 caused by the STAG2 c.462+1G>A variant.

**Figure S8**. Data from sample HN26, corresponding to a HNPGL from the sister of patient HN18 who carries the *SDHC* epimutation. (A) Sanger sequencing of a somatic *STAG2* frameshift variant identified in sample HN26, compared to the wild-type sequence observed in blood sample from the patient. (B) Positive SDHB IHC of sample HN26. (C) Principal component analysis of transcriptomic data from the sample HN26 and other HNPGLs. To facilitate data normalization, the HN13 sample and one *SDHD*-mutated control were sequenced alongside the HN26 sample. The HN26 sample exhibited a DNMT3A-like profile, close to the other *STAG2*-mutated sample, and separated from her sister’s sample, HN18. PCR amplification and Sanger sequencing of the promoter region of *SDHC* in bisulfite-modified DNA from sample HN26 (data not shown) demonstrated the absence of methylation. Considering all these data, the occurrence of two sisters with HNPGL in this family is a phenocopy rather than an inherited disease.

**C**

SDH-like

DNMT3A-like

SDHD

New samples

HN26 (blood sample)

HN26

HN26

**B**

**A**


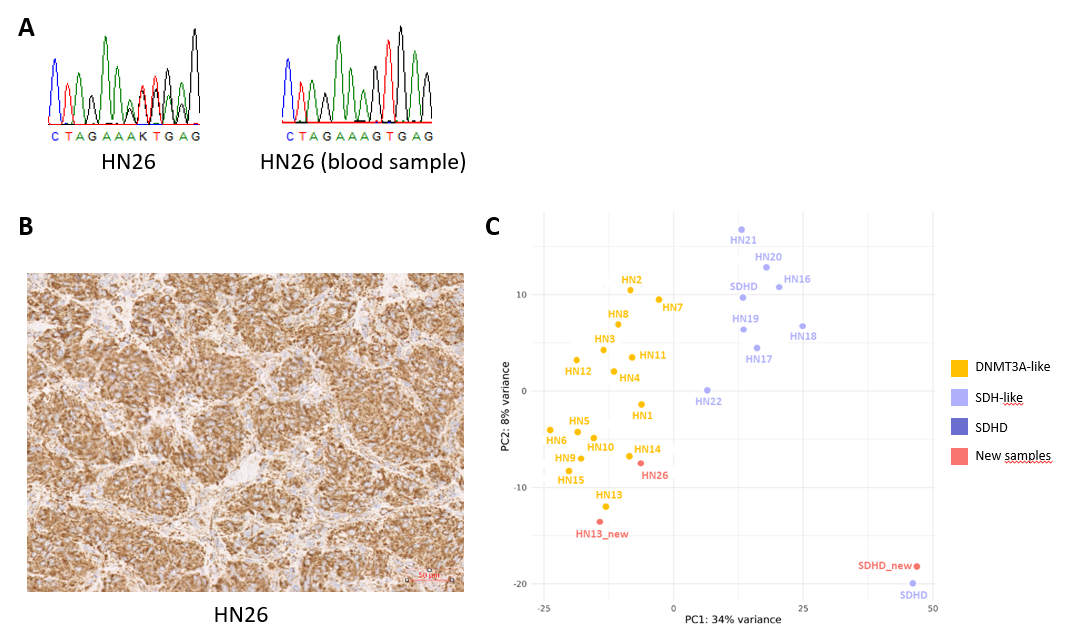

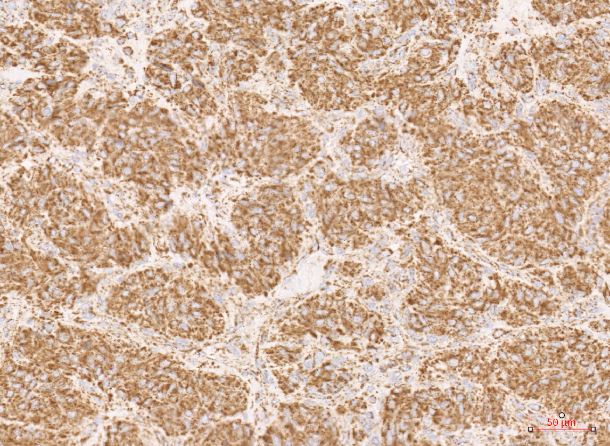

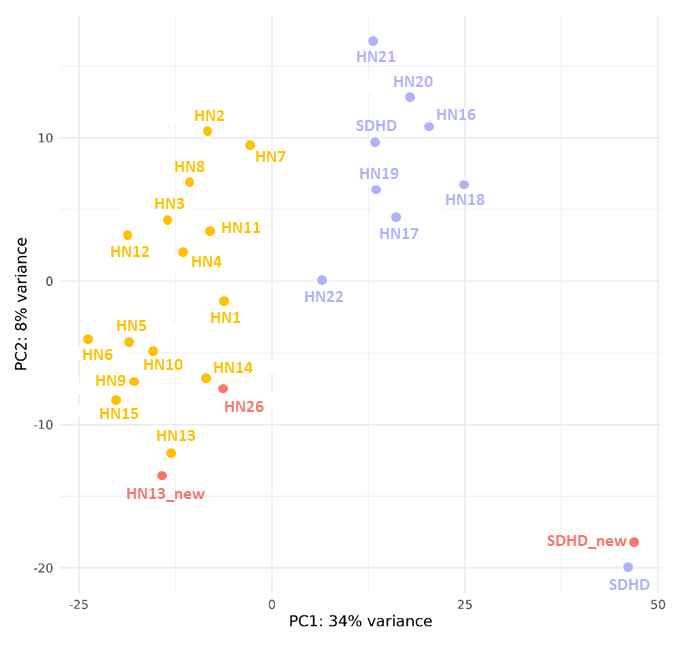


**Figure S9**. STAG2 IHC of tumors carrying *STAG2* mutations compared to a *STAG2* WT control (left). The lack of staining in the tumor cells was internally validated by the positive immunostaining observed in the sustentacular normal cells, indicated by black arrows. * New sample collected from a sister of patient HN18.


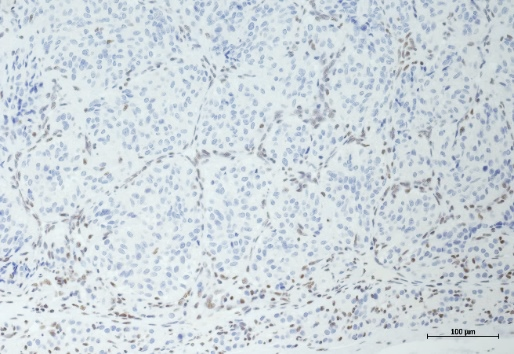

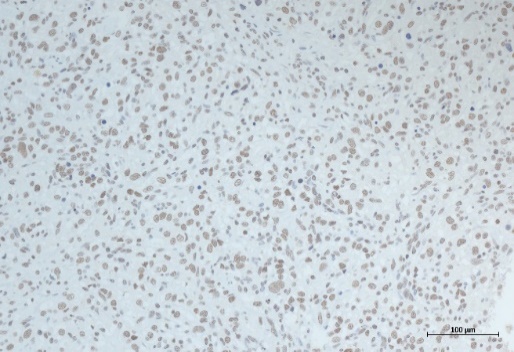

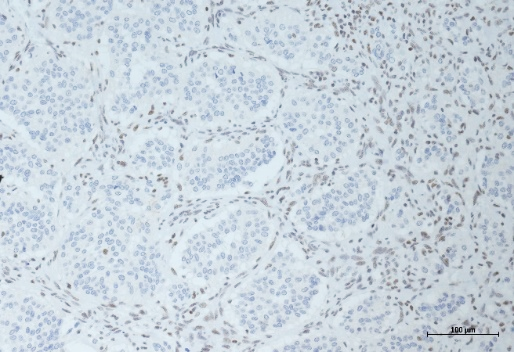


HN14 (*STAG2* c.462+1G>A)

*C+*

HN26* (*STAG2* p.Ser471Valfs*22)

STAG2 IHC


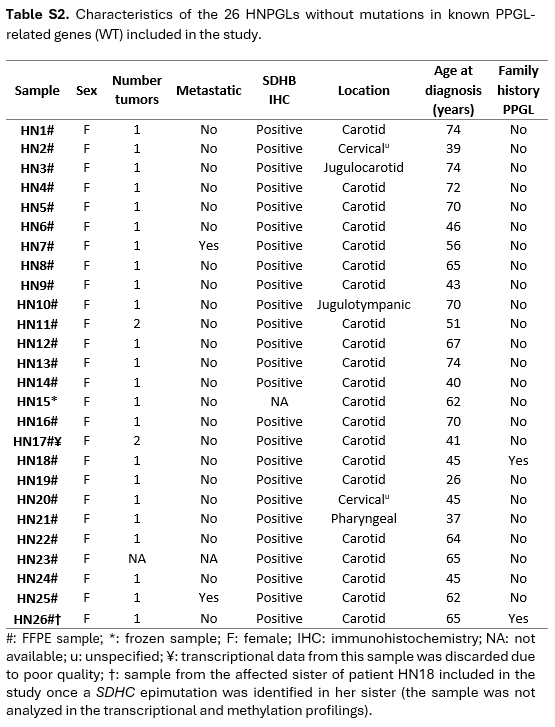

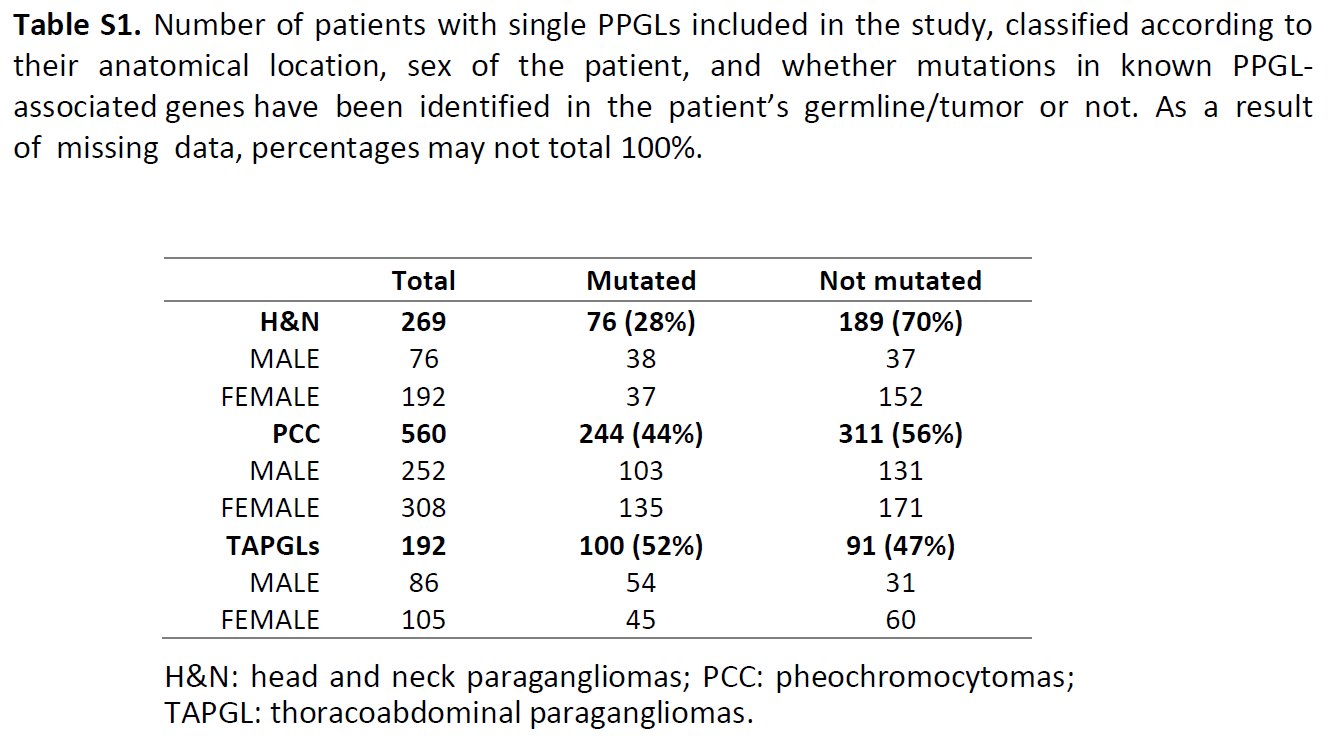


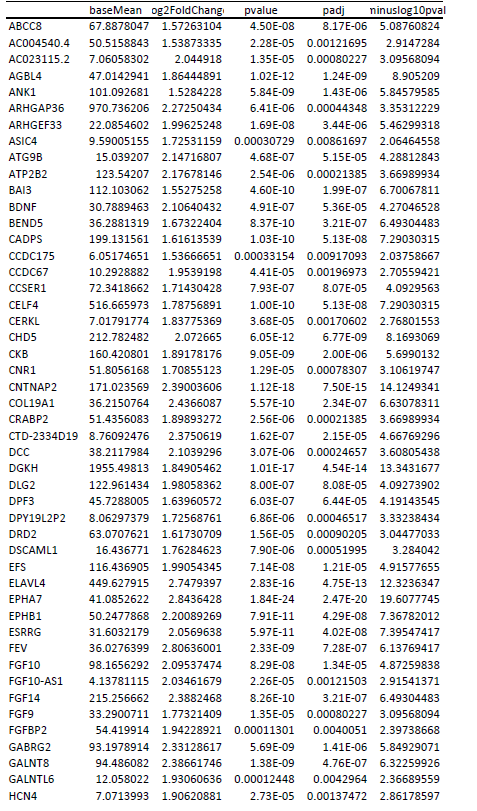


**Table S3**. List of 182 differentially expressed genes between DNMT3A-like and SDH-like WT HNPGLs (FDR < 0.01, log2FC < -1.5 or log2FC >1.5).


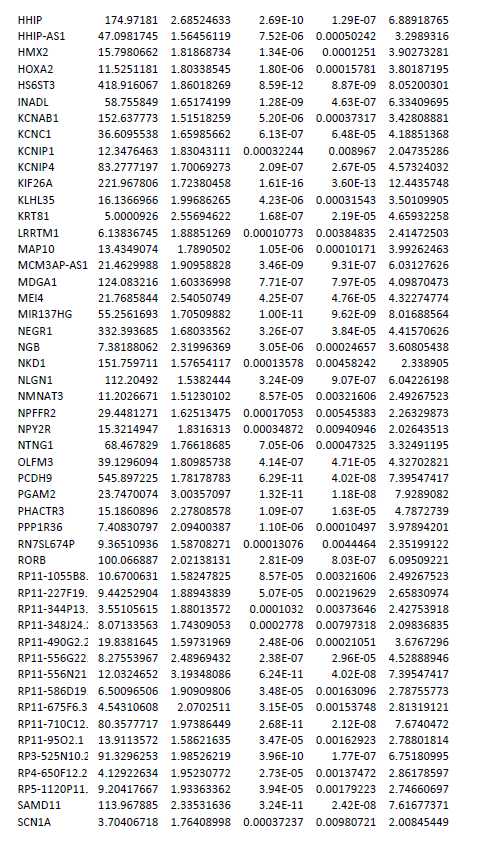


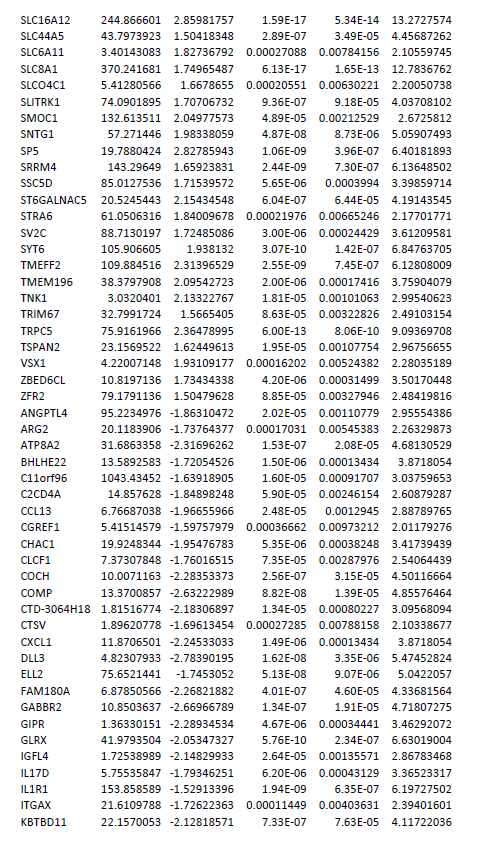


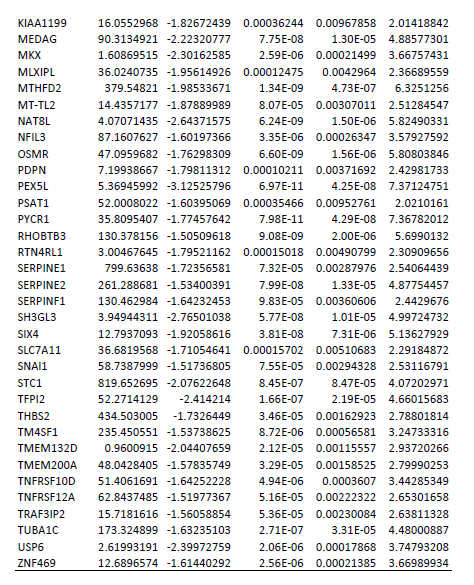


**Table S3**. List of 182 differentially expressed genes between DNMT3A-like and SDH-like WT HNPGLs (FDR < 0.01, log2FC < -1.5 or log2FC >1.5).

**Table S4**. Genomic alterations identified in WT HNPGLs.

*: sample HN26 corresponds to the affected sister of patient HN18 included in the study once a *SDHC* epimutation was identified in her sister (this sample was not analyzed in the transcriptional and methylation profiling).
